# Can Fecal T3 Metabolite Level Fluctuations in European Roe Deer (*Capreolus capreolus*) Give Insights on Body Condition and Thermal Stress?

**DOI:** 10.1111/1749-4877.12953

**Published:** 2025-02-07

**Authors:** Valeria Pasciu, Roberta Chirichella, Francesca D. Sotgiu, Maria Nieddu, Elena Baralla, Marco Apollonio, Fiammetta Berlinguer

**Affiliations:** ^1^ Department of Veterinary Medicine University of Sassari Sassari Italy; ^2^ Department of Humanities and Social Sciences University of Sassari Sassari Italy; ^3^ Department of Medicine, Surgery and Pharmacy University of Sassari Sassari Italy

**Keywords:** FTMs, individual and environmental variables, roe deer

## Abstract

Mammals can use a variety of physiological mechanisms to adapt to changes in their environment. Thyroid hormones (THs) are key modulators of growth and mediators of environmental conditions by regulating developmental processes and metabolism in animals. In recent years, advancements in non‐invasive sampling have allowed monitoring of the fluctuations of THs and their metabolites in wild mammals. Triiodothyronine (T3) represents the major metabolite of THs excreted in feces so that it can be monitored in fecal samples. In this study, fecal samples collected during the hunting season from legally culled European roe deer (*Capreolus capreolus*; *n* = 160) were assayed to investigate the potential fluctuations of fecal TH metabolites (FTMs) in response to environmental (e.g., the temperature, local densities) and individual (e.g., sex, age, body, and nutritional conditions) variables. For this aim, we validated a TH enzyme immunoassay in the feces of roe deer. Our results show that FTMs can be successfully measured with satisfactory accuracy and precision. Extraction recovery (70%–120%), intra‐ and inter‐day repeatability (<15%), linearity dilutions (80%–120%), and parallelism (<20%) were consistent with international guidelines. Environmental temperature (*p* < 0.001) showed a strong inverse correlation with FTM levels. THs can thus represent a reliable indicator in studying animals’ adaptative responses to environmental temperature changes, providing perspectives for the study of the impact of climate change on ungulates and mammals. Further analyses, comparing samples collected all year round, are needed to investigate the correlations of TH values versus the other investigated variables.

## Graphical abstract

European roe deer fecal samples were assayed to investigate the potential fluctuations of fecal thyroid hormone metabolites (FTMs) in response to environmental (e.g., the temperature, local densities) and individual (e.g., sex, age, body, and nutritional conditions) variables. Our results show that environmental temperature (*p* < 0.001) strongly correlated with FTM levels. FTM analysis could represent an indicator in studying the impact of climate changes on animals’ adaptative responses.

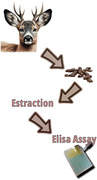

## 1 Introduction

The need to monitor wild ungulate populations and their condition is growing as a consequence of the importance of these species in the ecosystem and their relevance for human interests (Morellet et al. 2011; Carpio, Apollonio, and Acevedo 2021). An urgent challenge is to anticipate the effects of novel future conditions on their abundance and distribution to shape proper management strategies (Stillman et al. 2015). Classical strategies to address this issue use a top‐down approach, whereby demographic rates are related to environmental (e.g., temperature) or population‐level (e.g., density) variables. More recently, however, the responses to environmental changes are being assessed by the study of physiological variables at the individual level to predict population‐level effects in a bottom‐up approach (Johnston et al. 2019). Researchers’ efforts are thus focusing on the need to identify physiological markers, which give information about an individual's ability to cope with environmental harshness and are easily quantified in different biological matrices.

Thyroid hormones (THs) are key modulators of growth and mediators of environmental conditions by regulating developmental processes and metabolism in animals (Chatzitomaris et al. 2017; Hunninck et al. 2020; Mondol, Booth, and Wasser 2020; Houser et al. 2021; Pasciu, Sotgiu, et al. 2022; Pasciu, Nieddu, et al. 2022). The hypothalamus–pituitary axis regulates the secretion of THs, tetraiodothyronine (T4), and triiodothyronine (T3), through the thyrotropin‐releasing hormone (TRH), which stimulates the pituitary gland to secrete thyroid‐stimulating hormone (TSH) (Behringer et al. 2023). T4 is the predominant form of blood THs and displays a prolonged plasma half‐life, although it represents the inactive form (Sadoughi et al. 2021; Touitou et al. 2021). Despite having a minor half‐life, the circulating T3, deriving from T4 conversion at the peripheral level, is the more biologically active form (Chatzitomaris et al. 2017). THs have profound influences on metabolism, heart rate, blood pressure, nutritional physiology, brain development, and body temperature regulation independent of muscle activity (Dev, Sankar, and Vinay 2016; Pasciu, Sotgiu, et al. 2022; Behringer et al. 2023). They are also particularly responsive to nutritional deficits, lowering metabolism and allowing the body to conserve energy during a nutritional emergency (du Dot et al. 2009; Jesmer et al. 2017; Hunninck et al. 2020). Moreover, in homeotherms, their concentration is negatively correlated with external temperature as it is adjusted to maintain a constant body temperature when animals are outside their thermal neutral zone (Silva 2016; Pasciu, Nieddu, et al. 2022). THs can thus represent a key indicator in studying the responses of mammals to environmental stressors and the impact of climate changes on animal's adaptative responses. The predominant excretion of THs in bile offers the possibility of assaying their metabolites in feces, as demonstrated by several studies (Taurog, Briggs, and Chaikoff 1951; Wasser et al. 2010; Gesquiere et al. 2018). Recently, specifically in wild mammals (Wasser et al. 2010; Sadoughi et al. 2021; Touitou et al. 2021; Pasciu, Nieddu, et al. 2022; Pasciu, Molina‐López, and Baralla 2023), different methods have been used to monitor fecal TH metabolites (FTMs), of which the most abundant is T3 (Hunninck et al. 2020). Even in hunted individuals, feces represent easily retrievable matrices from the last tract of the rectum, within a few hours from the animal's death. On the contrary, collecting blood from dead animals immediately after shooting could be difficult in field‐hunting conditions, mainly because it would require the presence of specialized staff. However, despite providing a highly accessible, non‐invasive tool with enormous potential for understanding environmental physiology at individual and population levels, THs have received little attention compared to glucocorticoid and gonadal hormones (Wasser et al. 2010).

Starting from these premises, in the present study, we collected data from 160 European roe deer, legally culled during the 2022 hunting season (September–December period), in the Trento Province, Central‐Eastern Alps, Italy, to investigate the potential fluctuations of FTMs in relation to environmental (e.g., temperature experienced in the days before culling, local densities) and individual (e.g., sex, age, body, and nutritional conditions) variables.

The present study is part of a broader research aiming to investigate the condition of roe deer in the studied area to understand the reason for the decline of the species in the Central Alps. Roe deer experienced a tremendous increase in Europe after World War II and was considered a successful species (Andersen and Linnell 1998). However, in the last decades, a decrease was recorded in some European countries, and in the Alps, this reduction was quite pronounced and linked to environmental modifications (Chirichella, Mustoni, and Apollonio 2017). Contrasting evidence was found in analyzing the response of roe deer to climate change (Plard et al. 2013; Hagen et al. 2021). With this study, we aim to gain insight into the effect of the ecological context on roe deer physiological status, as evaluated by FTMs levels, to provide a new approach applicable to different wildlife species.

For this purpose, first, we provided the analytical and biological validation of an FTM assay in European roe deer feces. Then, we analyzed the relationships between environmental and individual variables and FTM concentrations of each roe deer examined.

## 2 Materials and Methods

### 2.1 Study Area and Species

**FIGURE 1:**
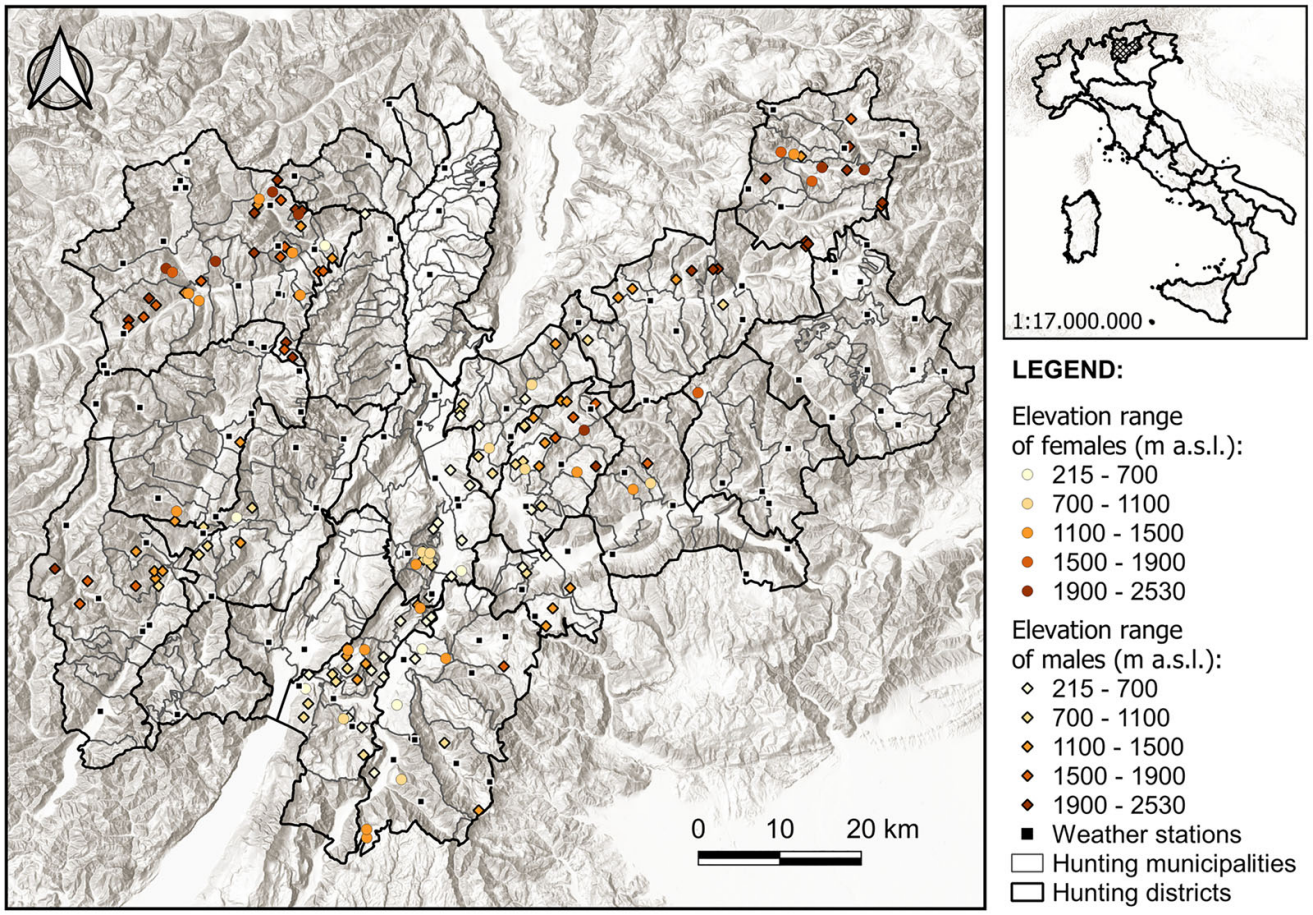
Map of the study area located in the Central‐Eastern Alps (6207 km^2^‐wide area; 46°04’N; 11°08’E; Province of Trento, Northern Italy) and consisting of 20 hunting districts subdivided into 209 municipal reserves (mean area: 310.50 km^2^, range: 155–622 km^2^) where 160 roe deer (*N*♀ = 42; *N*♂ = 118) were legally shot during 2022 hunting season (September–December period) are shown in the digital elevation model. The distribution of available weather stations (Forecasts and Organization Office‐Civil Protection Infrastructures Department of the Province of Trento, www.meteotrentino.it) was displayed.

The study area covered the Province of Trento in the Central‐Eastern Alps (6207 km^2^‐wide area; 46°04’N, 11°08’E; Northern Italy). Roe deer are managed in 20 hunting districts subdivided into 209 municipal reserves (mean area: 310.50 km^2^, range: 155–622 km^2^; Figure 1). Each hunted animal was registered by the Provincial Government. The hunters had hunting permits, and the hunting times/methods/hunting bags/zones were respected. The animals were not killed for research purposes. Therefore, the local ethics committee gave a favorable opinion on the use of post‐mortem animal material (protocol number 0066441). The climate varied between Alpine and semi‐continental climates. Annual precipitation amount was around 800 mm, while the temperatures varied between −5°C and −10°C in January and 20°C and 25°C in July (Forecasts and Organization Office‐Civil Protection Infrastructures Department of the Province of Trento, www.meteotrentino.it).

Elevations range from 65 m above sea level (a.s.l.) at the southern border around Lake Garda to 3558 m a.s.l. in the Presanella Massif. The area is forested up to the tree line at about 2000 m, above which it consists of Alpine meadows, rocky outcrops, scree fields, and open rock faces.

The European roe deer is a small‐sized cervid and is a solitary species, generally observed alone or in small family groups (Dzięciołowski 1979). Males exhibit territorial behavior from March to August, while females are not territorial, but are particularly solitary just prior to and following giving birth (Danilkin 1995) with little range overlap, even at high density (Maublanc et al. 2012). In the gregarious period (i.e., autumn and winter), group size is variable, particularly during the limiting season, and increases with landscape openness (Hewison et al. 2001), cover distribution (San José, Lovari, and Ferrari 1997), and density (Vincent et al. 1995). Roe deer is an income breeder and females that experience embryonic diapause mate in July–August and can produce on average two fawns (range 1–5) in May. It is a selective feeder and can live from sea level up to timberline. Roe deer can reach locally high densities of up to 30 deer/100 ha (Lorenzini et al. 2022).

The mean whole body mass of adult males ranges from around 18 to 32 kg, while adult females normally range from 17 to 30 kg (Lorenzini et al. 2022). Adult males are generally only 5%–10% heavier than females. Eviscerated body mass with head (without thoracic and abdominal organs) represents around 79%–80% of the live mass in adult bucks and does (Mattioli 2003). Hind foot length ranges from 35 to 40 cm while the length of the mandible is from around 145 to 167 mm (Lorenzini et al. 2022).

In the Trento province, roe deer experienced a strong increase after World War II but in the last two decades have been decreasing more or less markedly all over the area, possibly linked to a multifactorial array of reasons (Chirichella, Mustoni, and Apollonio 2017).

### 2.2 Sample Collection

We collected data from 160 roe deer (*N*♀ = 42; *N*♂ = 118) legally culled during the 2022 hunting season (September–December period; Figure 1). Date of culling, GPS localization, sex, and body weight (to the nearest 0.1 kg) were recorded by wardens. The body weight used in this study is eviscerated body mass (i.e., weighed without viscera and flowing blood).

For each culled animal, one of the hind foot were collected into plastic bags, sealed, marked, and stored at −20°C. Hunters also collected and cleaned mandibles using the hot water maceration method, hand removal of soft tissue and cartilage, and a 35% hydrogen peroxide treatment of all studied individuals for age assessment made by macroscopic inspection of teeth development and tooth wear (Ratcliffe and Mayle 1992; Chirichella et al. 2019, 2021, 2022) to evaluate the age class (juveniles: 1.5 y.o. animals; adults: ≥ 2.5 y.o. animals, related to the hunting period September–December). Each mandible was registered by the Provincial Government and made available to us for measurements.

In the laboratory, the hind foot (i.e., the distance from the top of the calcaneum to the tip of the hoof of the outstretched hind foot) length was measured with a digital caliper (to the nearest 0.01 mm) always by the same researcher. Body condition index (BCI) as the ratio of body mass to hind foot length was calculated for each animal.

Marrow samples were taken from the hind foot, placed in Petri dishes, weighed, and oven‐dried to constant weight at 80°C for 24 h, and their dry weight was expressed as a percentage of fresh weight (Neiland 1970; Gazzola et al. 2007; Bongi et al. 2008; Metz et al. 2012; Wilmers et al. 2020) and then categorized (three categories: low, medium and high nutritional status). Indeed, marrow fat percentage should be viewed as an indicator of fat, muscle, and energy depletion, and any level below a threshold of approximately 70%–85% should be taken to indicate generally poor conditions (Mech et al. 1995).

### 2.3 Environmental Variables

Considering the GPS localizations of each culled roe deer, we derived elevation values (m a.s.l.) and we associated each sample with the nearest weather station in the same altitudinal range. Averages of minimum, medium, and maximum temperatures (°C) experienced by each animal during the 7 days before culling were derived. Local density was estimated and then categorized (two categories [low: <0.8; good: ≥0.8]) by means of hunting culls on available habitat (heads/km^2^) in all municipal reserves from which we collected samples. Considering an average density of less than eight roe deer per 100 ha, hunting culls are derived from monitoring carried out with counts only where it is possible to observe roe deer during the spring period (i.e., monitoring to derive the minimum number of animals and, over the years, the population trend in more than 500 sampling areas with three annual repetitions), and data were associated with each municipal reserve (see Figure 1 for major details about municipal reserves descriptions).

### 2.4 Analyses of Fecal Samples and Validation of FTM ELISA Assay

#### 2.4.1 Collection and Storage of Fecal Samples

For each culled animal, fecal samples were collected from the rectum using a gloved hand. After sampling, they were immediately placed on ice (*<*45 min, median = 25 min) and within 4 h of culling (median = 1.5 h), stored at −20°C until further analysis (*<*1 year). These collection times have been applied in previous studies for fecal T3 hormone quantification (Hunninck et al. 2020) taking into consideration that previous studies have shown that thyroid hormone metabolite concentrations in feces stay stable (i.e., are not affected by bacterial degradation) within a similar time frame (2 weeks stored at 4°C, and 8 h stored at 25°C) (Gesquiere et al. 2018).

#### 2.4.2 Extraction of FTMs

For hormone extraction, fecal samples were defrosted at room temperature and individually homogenized. Wet feces (0.2 g of feces) were freeze‐dried in 15‐mL tubes. Then, FTMs were extracted following a protocol previously described by Pasciu et al. (Pasciu, Sotgiu, et al. 2022; Pasciu, Nieddu, et al. 2022). Briefly, the freeze‐dried samples were extracted with ethanol 70%, dried under a stream of compressed air, and the residues were reconstituted with 1 mL of phosphate‐buffered saline (PBS).

#### 2.4.3 FTM Quantifications by ELISA Assay

To determine FTM levels, we used an ELISA kit obtained from Diametra (DiaMetra Srl Management and Coordination: Immunodiagnostic Systems [IDS] Ltd, Boldon, UK, Perkin Elmer Company). Triiodothyronine (T3) standard and ethanol were obtained from Merck (Merck KGaA, Darmstadt, Germany; product cod. 642511 and 51976). Plates were read using a microplate reader (POLAR star Omega; BMG Labtech) with BMG Labtech software. The ELISA kit was previously validated for FTM quantification in mouflons (Pasciu, Nieddu, et al. 2022) and sheep (Pasciu, Sotgiu, et al. 2022). This competitive method, designed for human T3 assay in blood serum or plasma, is based on group‐specific polyclonal antibodies able to identify also its metabolites. The kit's lower detection limit was 0.05 ng/mL, suitable for our analytical purpose. The specific cross‐reactivity supplied by the manufacturer was: l‐triiodothyronine (1.00%), d‐triiodothyronine (0.015%), l‐thyroxine (0.01%), d‐thyroxine (0.0025%), monoido‐tyrosine (n.d.), diiodo‐tyrosine (n.d.), tri‐iodothyro‐acetic acid (n.d.), and tetra‐iodothyro‐acetic acid (n.d.). Quality controls provided by the manufacturer were used to verify the performance of the assay. T3 concentrations were calculated through a calibration curve ranging from 0 to 7.5 ng/mL.

#### 2.4.4 Validation of FTM Assay

To correctly interpret the variations in hormone concentrations, we validated the method used in Roe deer. Here, we conducted both an analytical validation, using standard assay validations of precision, accuracy, dilutional linearity and parallelism, and a biological validation, by assessing whether temperature variations affected FTM concentrations. We expected a negative relationship as THs are crucially important in thermogenesis and thermoregulation in homeotherms and they increase in concentration in response to cold temperatures (Silva 2016). Previous studies considered at this aim seasonal variations (Gesquiere et al. 2008; Chinnadurai et al. 2009; Hunninck et al. 2020).

For the analytical validation, basal FTM concentration was determined analyzing a pool of 10 roe deer feces. To assess accuracy and precision parameters, 0.2 g of feces was spiked with standard T3 at two levels (1 and 5 ng). Then, all samples were freeze‐dried and extracted with 70% ethanol using the method of Pasciu et al. (Pasciu, Sotgiu, et al. 2022; Pasciu, Nieddu, et al. 2022). Dry residues were reconstituted with 1 mL of PBS to obtain final concentrations of 1 and 5 ng/mL. The precision of the method, expressed as percent relative standard deviation (RSD%), was calculated for three replicates on the same day (intra‐day repeatability) and over three consecutive days (inter‐day repeatability). The accuracy, which represents the closeness of the test results to the true values, was determined as % extraction recovery, for five replicates, and calculated by comparing spiked samples at two different concentrations (deducted from basal level) with the correspondent theoretical concentration (1 or 5 ng/mL in PBS), using the following formula:

$$\%\;\mathrm{Extraction}\;\mathrm{recovery}=\frac{\left(\mathrm{Measured}\;\mathrm{concentration}\;\mathrm{spiked}\;\mathrm{sample}-\mathrm{Measured}\;\mathrm{concentration}\;\mathrm{unfortified}\right)}{\mathrm{Theoretical}\;\mathrm{concentration}}\;\times \;100$$

To assess the dilutional linearity, three serial dilutions of fortified samples were analyzed in duplicates, compensating for the dilution factor, and calculating the coefficient of variation (% CV) and % dilution recovery for each serial dilution (Interference, n.d.; Andreasson et al. 2015). Regarding % dilution recovery, it was calculated using the following formula (Interference, n.d.):

$$\begin{array}{ccc} & & \%\;\mathrm{Change}\;\mathrm{in}\;\mathrm{concentration}\;\mathrm{from}\;\mathrm{previous}\;\mathrm{dilution}\\ & & =\frac{\mathrm{Analyte}\;\mathrm{concentration}\;\;\mathrm{at}\;2^{\circ }\;\mathrm{dilution}}{\mathrm{Analyte}\;\mathrm{concentration}\;\;\mathrm{at}\;1^{\circ }\;\mathrm{dilution}}\;\times 100 \end{array}$$

$$\begin{array}{ccc} & & \%\;\mathrm{Change}\;\mathrm{in}\;\mathrm{concentration}\;\mathrm{from}\;\mathrm{previous}\;\mathrm{dilution}\\ & & =\frac{\mathrm{Analyte}\;\mathrm{concentration}\;\mathrm{at}\;3^{\circ }\;\mathrm{dilution}}{\mathrm{Analyte}\;\mathrm{concentration}\;\mathrm{at}\;2^{\circ }\;\mathrm{dilution}}\;\times 100 \end{array}$$

Dilutional linearity is performed to demonstrate that a sample, with a spiked concentration above the upper limits of quantification, can be diluted to a concentration within the working range and still give a reliable result (Andreasson et al. 2015). To verify whether the assay maintained linearity when unfortified samples were diluted, we performed parallelism, using non‐spiked with high endogenous concentrations of the analyte. Three serial dilutions for each non‐spiked sample were analyzed in duplicates, by compensating with the dilution factor. Parallelism was expressed as % CV.

### 2.5 Statistical Analysis

GLM was calculated between FTM levels and individual (sex, age [juveniles vs. adults], BCI, nutritional status [low, medium, high]) and environmental (temperature, elevation, and local density [low vs. good]) variables. Variables were scaled and models were compared by means of Akaike's information criterion for small sample sizes (AICc). We checked standardized residual plots for assumptions of normality, homoscedasticity, and independence (Burnham and Anderson 2002; Zuur et al. 2010). We obtained the effect of each variable (i.e., parameter estimation) included in the confidence set of models via model averaging (model. Avg function in MuMin package applied to ΔAICc ≤ 6; (Burnham and Anderson 2002; Symonds and Mousalli 2011). Statistical analyses were performed using R version 4.0.4 (www.r‐project.org; (R Core Team 2013)).

## 3 Results

In this study, FTMs of 160 wild ungulates (118 males; 42 females), European roe deer, were assayed to investigate the potential fluctuations of FTMs in response to environmental (e.g., the temperature, local densities) and individual (e.g., sex, age, body, and nutritional conditions) variables. Adult males (*N* = 112; mean age = 3.13 y.o.) showed a mean eviscerated body mass of 17.92 kg (juveniles = 17.61; *N* = 6) and a BCI of 0.54 (juveniles = 0.53; *N* = 6), while the nutritional status was 72% (juveniles = 76%; *N* = 6). Adult females (*N* = 31; mean age = 3.77 y.o.) showed a mean eviscerated body mass of 17.01 kg (juveniles = 16.99; *N* = 11) and a BCI of 0.52 (juveniles = 0.52; *N* = 11), while the nutritional status was 81% (juveniles = 88%; *N* = 11).

### 3.1 Validation of FTM Assay

Accuracy values, expressed as average recovery on fecal samples (Table 1) were 82.11% and 106.21% for samples spiked with 1 and 5 ng/mL, respectively. The intra‐day and inter‐day repeatability expressed as RSD % were within 15% for both spiked concentrations (Table 1).

**TABLE 1:** Repeatability and recovery in fecal samples at two concentration levels.

Sample	T3 spiked (ng/mL)	Repeatability (RSD%) Intra‐day Inter‐day	Recovery (% ± SE)
**Feces**	1.0	4.97 13.93	82.11 ± 10.93
	5.0	7.51 14.45	106.21 ± 11.72

Abbreviations: RSD% = relative standard deviation; SE = standard error.

The dilution recovery was between 89% and 118%, and parallelism (% CV) was <20%. The parallelism curve was reported in Supporting information (Figure S1). Analytical validation parameters showed results in accordance with the guidelines for validation of an ELISA method (Andreasson et al. 2015).

**FIGURE 2:**
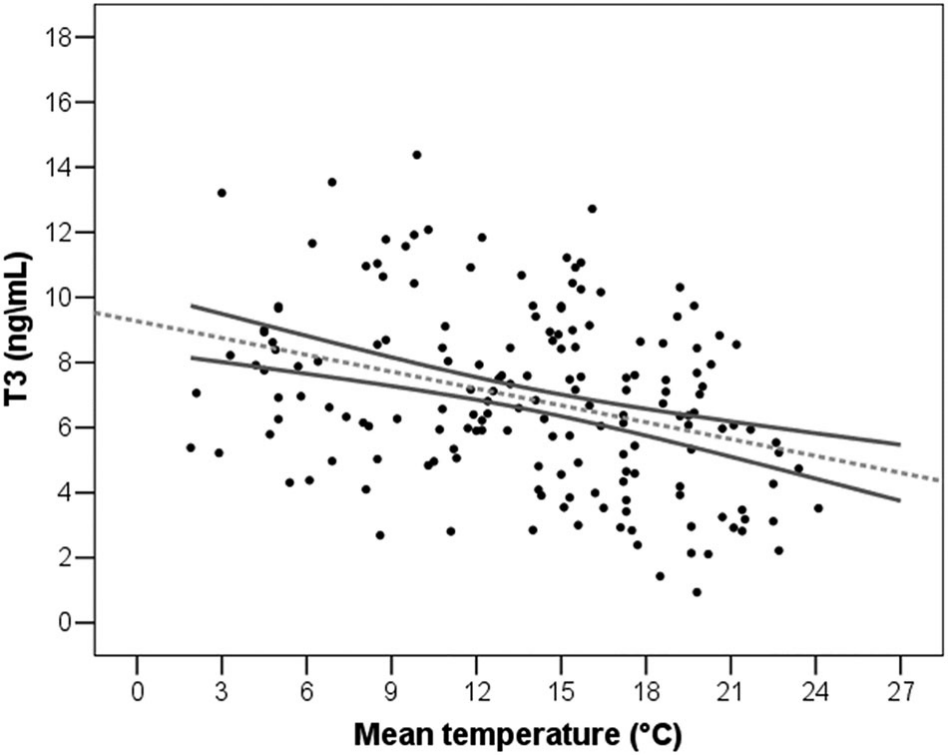
Variation in T3 concentrations (ng/mL) in 160 roe deer (*N*♀ = 42; *N*♂ = 118) legally shot in the Province of Trento (Northern Italy) during the 2022 hunting season (September–December period) in relation to increasing mean temperature (°C) experienced by animals during the 7 days before culling. Linear regression (gray dotted line) and 95% confidential Intervals (gray continuous lines) were reported.

As for the biological validation, the mean value of the FTMs (6.763 ± 2.726 ng/mL) measured was consistent with those we found in previous works in wild and domestic ungulates (Pasciu, Sotgiu, et al. 2022; Pasciu, Nieddu, et al. 2022; Pasciu, Molina‐López, and Baralla 2023). As expected, FTM concentrations significantly varied in response to the mean temperature experienced by animals 7 days before culling (*p* < 0.001), with higher levels in response to lower temperatures (Figure  2).

### 3.2 Model Results

We compared FTM levels to different studied variables: age (juveniles vs. adults), sex, BCI, nutritional status, and temperature experienced by animals during the 7 days before culling (range of mean daily temperature: 1.9–24.1°C), elevation, and local densities. Only the mean temperature had a significant effect on the FTM levels (*p* < 0.001) (i.e., higher value in response to a lower temperature; see Figure 2). However, in our dataset, males had higher FTM levels, as well as roe deer in better body conditions even if this trend was not statistically significant (Table S1). According to environmental variables, a negative effect of elevations and local densities on FTM levels was revealed. The results obtained were reported in Tables 2 and 3.

**TABLE 2:** Set of the most parsimonious models (ΔAIC and ΔAICc <6) showing variation in FTMs concentration (ng/mL) of 160 roe deer in Trento Province, Centra‐Eastern Alps, Italy.

Component models	AIC^a^	ΔAIC^a^	AICc^b^	ΔAICc^b^
Sex + BCI + T mean	299.393	0	755.843	0
Sex + BCI + T mean + Elevation	300.871	1.478	757.481	1.638
Sex + BCI + Age class + T mean + Elevation	302.752	3.359	759.549	3.706
Sex + BCI + Age class + T mean + Elevation + Local density	304.749	5.356	761.763	5.920

^a^ Akaike's information criterion. ^b^ Akaike's information criterion for small sample size.

**TABLE 3:** Parameter estimates of averaged linear models predicting FTM concentration (ng/mL) of 160 roe deer in Trento Province, Centra‐Eastern Alps, Italy.

Parametric coefficients	Estimate	SE	Adj. SE	*Z*	*p*
Intercept	6.273	0.404	0.408	15.392	<0.001
Sex [male]	0.669	0.471	0.475	1.410	0.159
Body Condition Index	0.293	0.207	0.208	1.409	0.159
Age class [juveniles]	−0.237	0.703	0.709	0.335	0.738
Temperature (mean)	−1.131	0.210	0.211	5.351	≤0.001
Elevation	−0.158	0.216	0.218	0.723	0.470
Local density	−0.021	0.422	0.425	0.049	0.961

Descriptive statistics about the FTM levels in response to the most important individual and environmental variables were reported in Table S1.

## 4 Discussion

In this work, we analyzed the fluctuation of FTMs in response to individual and environmental variables in the roe deer. In detail, sex, age (juveniles vs. adults), BCI, and nutritional status (low, medium, high) for individuals and temperature, elevation, and local density (low vs. good) for environmental variables, were considered.

No differences in FTMs were found between male and female roe deer. The difference in FTMs between male and female individuals is not consistent across studies performed in several species, as well as ungulates (Pasciu et al. 2024); in some studies, no difference is found between sexes (Hunninck et al. 2020; Pasciu, Nieddu, et al. 2022; LaDue et al. 2023), while in others, FTM concentration was higher in males than females, or vice versa (Hu et al. 2018; Mondol, Booth, and Wasser 2020; Houser et al. 2021). These outcomes regarding sex could be probably explained considering the overriding influence of other variables on hormonal levels (Suzuki et al. 2012; Pasciu et al. 2024).

Regarding age, different authors reported that young animals have higher TH levels than adults, and this finding is consistent both for TH concentrations in blood and those of their metabolites in feces (Novoselec et al. 2016; Hunninck et al. 2020; Pasciu, Sotgiu, et al. 2022). This has also been observed in wild ungulates (e.g., forest musk deer, *Moschus brezowski*), where higher FTM levels were found in young individuals than in adults and decreased as they grew up (Hu et al. 2018). Age‐related differences in TH concentrations are well described in domestic ungulates, with the highest values found in neonates and the lowest in elderly animals (Todini 2007; Pasciu, Sotgiu, et al. 2022). These data are explained by the TH action in controlling metabolism that, during the growing period, especially immediately after birth, must be higher to promote the individual's growth (Hu et al. 2018; Pasciu, Sotgiu, et al. 2022). In our study, we found no differences between the FTMs of adult versus juvenile roe deer, probably because juvenile‐hunted roe deer had almost completed their development, being on average 16 months old and less than 20% lighter than adults. Moreover, environmental conditions might also force adults to have high T3 values like those of juveniles, as low temperature can increase T3 in adults reducing the possible age gap (Reed 1995). This hypothesis needs to be tested in roe deer, by further studies with younger individuals and including samples collected in the warmer months of the year.

We considered the BCI and nutritional status as potentially influencing FTMs, the latter being particularly responsive to nutritional deficits (Hunninck et al. 2020). In fact, in literature lower levels of FTMs are observed when food intake is low as well as during periods of dietary reduction, fasting, or hibernation (Behringer et al. 2018; Hunninck et al. 2020; Frare, Williams, and Drew 2021). Low FTM values indicate energy conservation, which results in slow metabolism (Behringer et al. 2018; Hunninck et al. 2020), probably as an adaptive response to changes in food quantity and quality (Hu et al. 2018). However, in this study, no fluctuation of FTMs was found in relation to the BCI and nutritional status. Samples were collected between September and December when animals were probably beginning to adapt their metabolism to a decrease in food availability. While nutritional status had higher values in females than males, BCI did not show major variations between sex and age classes. This aspect deserves to be further investigated by monitoring, over a year, the eventual fluctuation of FTMs concerning BCI and nutritional status.

Among the analyzed variables, temperature showed a negative correlation with increasing FTMs. This is in accordance with other authors for macaques (*Macaca sylvanus*) (Cristóbal‐Azkarate et al. 2016; Silva 2016), llamas (*Lama glama*), burros (*Equus asinus*) (El‐Nouty et al. 1978), impala (*Aepiceros melampus*) (Hunninck et al. 2020), sheep (*Ovis aries*) (Novoselec et al. 2016), mouflons (*Ovis gmelini musimon*) (Pasciu, Nieddu, et al. 2022), and elephant (*Loxodonta africana*) (Szott et al. 2020), where significantly higher FTM levels were found when temperature decreased and an increase in temperature was associated with a decrease in FTMs. Since thyroid hormones are known to have a critical role in the thermoregulation mechanism in homeotherms, our results confirm that fluctuations in FTM levels are correlated with changes in environmental temperature. This result opens wide perspectives on the use of FTM fluctuation to study the impact of climate changes on ungulates and mammals in general, an issue that nowadays deserves particular attention (Post et al. 2009; Radchuk et al. 2019).

Despite our expectations, population density was not significantly related to FTM levels in the studied population. This finding could be correlated to the low/medium levels (mean value: 7.5 animals/km^2^; range: 3–16 animals/km^2^) of the local density which does not generate a density‐dependent effect.

## 5 Conclusion

In this study, we performed an analytical and biological validation of an ELISA method to measure accurately T3 metabolite concentrations in roe deer feces for the first time. Obtained results showed that only environmental temperature, among all the studied variables, caused FTM fluctuations by increasing them as temperature decreased.

These results confirm that thermoregulation may be a critical aspect of TH regulation and FTMs can provide insights into this frame, while body condition influences only partly the FTM fluctuations in roe deer. Overall, we showed how a non‐invasive matrix such as feces can provide important information about the roe deer's physiology. Moreover, the validated method could be useful for future studies on energy expenditure, seasonality, and pathological conditions of roe deer and other wild ungulates.

The lack of further correlations with environmental and individual parameters deserves further studies based on a more variable dataset and longitudinal data.

## Conflicts of Interest

The authors have no conflicts of interest to declare.

## Supporting information




**Figure S1** Comparison of standard vs parallelism curves.
**Table S1** Faecal T3 metabolites (FTMs) levels (ng/mL) of 160 roe deer in Trento Province, Centra‐Eastern Alps, Italy. [* = variables categorized only for data description in this supplementary material but inserted in model selection as continuous variables].
