# Supplementary material for: Can Fecal T3 Metabolite Level Fluctuations in European Roe Deer (Capreolus capreolus) Give Insights on Body Condition and Thermal Stress?
Source: Integr Zool. 2025 Feb 7;21(3):552–60. doi: 10.1111/1749-4877.12953 (PMC13165694; doi:10.1111/1749-4877.12953)
Supplement: Supplementary file 1 — Figure S1 Comparison of standard vs parallelism curves. Table S1 Faecal T3 metabolites (FTMs) levels (ng/mL) of 160 roe deer in Trento Province, Centra‐Eastern Alps, Italy. [* = variables categorized only for data description in this supplementary material but inserted in model selection as continuous variables]. [file INZ2-21-552-s001.docx]

# SUPPLEMENTARY MATERIALS


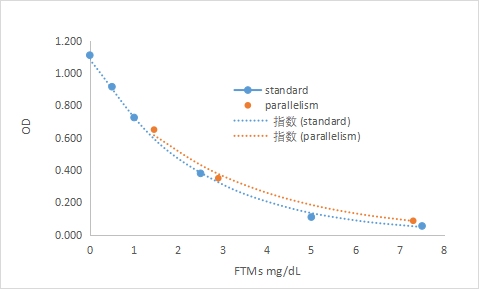


**Figure S1** Comparison of standard vs parallelism curves.

**Table S1** Faecal T3 metabolites (FTMs) levels (ng/mL) of 160 roe deer in Trento Province, Centra-Eastern Alps, Italy. [*= variables categorized only for data description in this supplementary material but inserted in model selection as continuous variables].

|  |  | **T3 (ng/mL)** | | | |
| --- | --- | --- | --- | --- | --- |
| **Sex** | **Age class** | **Mean** | **SD** | **Minimum** | **Maximum** |
| **Females** | Adults | 6.498 | 3.253 | 0.935 | 13.543 |
|  | Juveniles | 7.022 | 1.506 | 4.308 | 8.992 |
|  | **Total** | **6.635** | **2.890** | **0.935** | **13.543** |
| **Males** | Adults | 6.822 | 2.629 | 1.428 | 14.385 |
|  | Juveniles | 6.548 | 3.751 | 2.841 | 13.213 |
|  | **Total** | **6.808** | **2.676** | **1.428** | **14.385** |
| **Total** |  | 6.763 | 2.726 | 0.935 | 14.385 |

|  | **T3 (ng/mL)** | | | |
| --- | --- | --- | --- | --- |
| **BCI (kg/cm) *** | **Mean** | **SD** | **Minimum** | **Maximum** |
| **Low (<0.4)** | 6.497 | 2.546 | 2.220 | 11.837 |
| **Medium (0.4-0.6)** | 6.756 | 2.740 | 0.935 | 13.543 |
| **High (>0.6)** | 7.030 | 2.901 | 1.428 | 14.385 |
| **Total** | 6.763 | 2.726 | 0.935 | 14.385 |

|  | **T3 (ng/mL)** | | | |
| --- | --- | --- | --- | --- |
| **Elevation (m asl) *** | **Mean** | **SD** | **Minimum** | **Maximum** |
| **Low (<800)** | 6.973 | 2.461 | 2.145 | 12.718 |
| **Medium (800-1800)** | 6.713 | 2.716 | 0.935 | 14.385 |
| **High (> 1800)** | 6.666 | 3.054 | 2.389 | 13.543 |
| **Total** | 6.763 | 2.726 | 0.935 | 14.385 |

|  | **T3 (ng/mL)** | | | |
| --- | --- | --- | --- | --- |
| **Local density (heads/km^2^)** | **Mean** | **SD** | **Minimum** | **Maximum** |
| **Low (0.1-0.8)** | 6.720 | 2.811 | 0.935 | 14.385 |
| **Good (0.9-3.1)** | 6.815 | 2.637 | 1.428 | 13.213 |
| **Total** | 6.763 | 2.726 | 0.935 | 14.385 |

|  | **T3 (ng/mL)** | | | |
| --- | --- | --- | --- | --- |
| **Nutritional status (%)** | **Mean** | **SD** | **Minimum** | **Maximum** |
| **Low (<50%)** | 6.362 | 2.919 | 2.220 | 9.737 |
| **Medium (50-75)** | 7.010 | 2.813 | 2.389 | 11.785 |
| **High (> 75%)** | 6.721 | 2.707 | 0.935 | 14.385 |
| **Total** | 6.763 | 2.726 | 0.935 | 14.385 |

|  |  | **T3 (ng/mL)** | | | |
| --- | --- | --- | --- | --- | --- |
| **Temperature (°C) *** | **Sex** | **Mean**  **[Juveniles; Adults]** | **SD**  **[Juveniles; Adults]** | **Minimum**  **[Juveniles; Adults]** | **Maximum**  **[Juveniles; Adults]** |
| **1.9≤T<9.3** | Females | 8.145  [7.141; 8.693] | 2.807  [1.602; 3.223] | 4.308  [4.308; 4.385] | 13.543  [8.685; 13.543] |
|  | Males | 7.920  [8.822; 7.209] | 2.401  [4.122; 2.095] | 4.096  [5.034; 2.692] | 13.213  [13.213; 10.642] |
|  | Total | 7.755 | 2.579 | 2.692 | 13.543 |
| **9.3≤T<16.7** | Females | 6.305  [7.768; 5.968] | 2.626  [1.107; 2.785] | 2.806  [1.107; 2.785] | 11.922  [6.836; 2.806] |
|  | Males | 7.752  [6.045; 7.785] | 2.580  [-; 2.594] | 3.005  [6.045; 3.005] | 14.385  [6.045; 14.385] |
|  | Total | 7.417 | 2.643 | 2.806 | 14.385 |
| **16.7≤T≤24.1** | Females | 4.380  [5.547; 4.033] | 1.813  [1.140; 1.190] | 0.935  [4.742; 0.935] | 6.353  [6.353; 6.144] |
|  | Males | 5.370  [3.387; 5.464] | 2.312  [0.773; 2.322] | 1.428  [2.841; 1.428] | 10.314  [3.934; 10.314] |
|  | Total | 5.200 | 2.252 | 0.935 | 10.314 |
| **Total** |  | 6.763 | 2.726 | 0.935 | 14.385 |
